# Round the Hospitals

**Published:** 1920-01-17

**Authors:** 


### ROUND THE HOSPITALS.

We learn with pleasure that Queen Alexandra has graciously accepted from the Council of the College of Nursing a copy of the first register of its members.

DR. ELIZABETH SLOAN CHESSER addresses a letter to the Press appealing to girls who are being demobilised from military and Government Services to take a year's free training under the County

**ROUND THE HOSPITALS—(*continued*).**

Nursing Association "nurse midwives." It is a very good letter and we trust many women may be attracted by it to the work she describes. But we desire to call attention to the misuse of the word *nurse* in this connection. Parliament has just consented to pass a measure which by universal consent will result in the fixing of a minimum standard of three years' hospital training for nurses. To this minimum nurses must add from four to six months' training in midwifery before they are fully qualified as nurse and midwives. How can these things be made to harmonise with one year's training? Cannot the nursing world be delivered from the self-contradictory term "nurse" to express a registered and certificated woman, in view of the fact that it is also taken to mean (1) a probationer; (2) a midwife; (3) an amateur assistant; (4) an attendant; and (5) a maid-servant? We should like to see either some authoritative pronouncement that a few weeks' extra instruction will not turn a midwife into a nurse, or (better) that the vague term "nurse" had been definitely abandoned in favour of some more explicit description for women placed on the registrar. Some of our readers may have noticed on the same page of *The Times* on the 9th inst. two head-lines—"Nurse charged with shop-lifting," "Nurse charged with cruelty." One of these was a V.A.D. member, the other a maid-servant.

---

Lord Knutsford exposes a great blot on the private nursing system when he writes of the nurses who for twenty years have been undertaking cases requiring twelve hours' vigilant attendance on the sick. For several weeks, he states, after a severe operation he was never left alone by his two nurses. He claims that they were never exhausted, but a moment's consideration will show that at times during every day both must have been severely overstrained, though they did not show signs of it to the patient. Conditions such as he describes call in common humanity for three nurses, who even then, reckoning duplication at relief times, would be on duty some nine hours a day each. District nurses should be required to do eight hours' work a day as an average routine, but longer hours in view of distance may be unavoidable. Only emergency work on Sundays is desirable.

---

Many of our readers will be glad to hear that the Brompton Hospital for Consumption is continuing its admirable courses for trained nurses, health visitors, tuberculosis visitors, and social workers during February and March. The course comprises seventeen lectures on tuberculosis, illustrated with the aid of the epidiascope; it covers practical instruction in the laboratory and in the wards of the hospital, and also affords opportunities for seeing dispensary practice in the out-patient department, of visiting dispensary patients under the charge of the dispensary sister and of inspecting the sanatorium at Frimley. It is in fact by far the most complete form of post-graduate instruction for nurses in the ramifications of this disease which has yet been thought out. The fee for lectures alone is £1 1s.; that for the complete course, £2 2s., with an optional examination. The opening lecture on the "History of Tuberculosis" will be given by Dr. L. S. Burrell, on Tuesday, February 17, at 8 P.M.

---

Increased prices dominate the nursing problems to an extent very little recognised. The general rise in salaries is to some extent delusive even in institutions, for the claims on the nurse's purse have doubled and trebled. In district, school and other extern forms of nursing the rise in salaries has by no means kept pace with that in the cost of living, so that thrifty women find they have far less spare money than they used to have. Meantime the cost of midwifery training—always a heavy charge on nurses—is mounting up. In the Military Families' Hospital the fee for board payable by nurses who are admitted to a four months' course of midwifery training has just been raised fifty per cent., and now stands at fifteen guineas instead of ten. This brings it to about 18s. a week.

---

The Westminster Union Infirmary at Hendon has been purchased by the Metropolitan Asylums Board at an agreed price of £160,000. It will be used for the accommodation of tuberculous patients, for which its fine position makes it eminently suitable. The Board has agreed to take over the staff employed at the Infirmary with the exception of the medical superintendent, the probationer nurses, and certain other officers remaining in the service of the Guardians. The hospital will accommodate some 300 cases. Particulars with regard to the training or part-training of the nurses will be awaited with interest.

---

There are many institutions in which the managers are painfully aware that the least satisfactory of their departments is the laundry. This is largely due to defective installation of machinery, which may be responsible for the fact that a laundry which should afford a considerable saving is continually showing a deficit, compared with the cost as estimated. Expert assistance in planning new laundries and installing modern apparatus will save many blunders, and we have pleasure in bringing to our readers' notice the name of Mr. H. S. Wood, Consulting Engineer, 67 Deodar Road, Putney, S.W. 15, who is at present advising the Spalding Guardians, the Sudbury Guardians, and the Committee of the Swansea General Hospital. It is worth remembering that comparatively small alterations may turn the laundry from a failure to a success.

---

Next to the importance of adequate salaries must be reckoned the shortening of duty hours. At the Royal Southern Hospital an extension to the Nurses' Home is already nearing completion which will provide eighteen more bedrooms, six more bath-

**ROUND THE HOSPITALS**—(*continued*).

rooms and three housemaids' closets. The best models have been followed in this extension. As far as possible there will be fixed furniture. Shampoo basins with fittings are being included, hot pipes will be utilised for airing linen, and cloakrooms are provided for changing wet outer garments. The nursing staff of the hospital have a great advantage in the existence of a Nurses' Holiday and Rest Fund from which, as occasion arises, grants are made to enable members of the staff to take a good holiday and enjoy a rest free from financial worries after illness. The scope of the Fund is extended to cover cases in which nurses cannot afford to take courses of training, such as midwifery or massage, without help. When the extension to the Nurses' Home is completed all the nurses will enjoy one day's rest in seven. The consideration shown by the authorities for the nurses' well-being cannot fail to react most favourably on this first-class training-school. The scheme reflects the highest credit on the matron, Miss Agnes Bagnall.

---

It is a great testimony to the high place which nursing has now secured in public estimation that Mr. Joseph Watson, of Linton, Springs, Wetherley, should have earmarked no less a sum than £10,000 out of his munificent New Year's gift to the Leeds General Infirmary to be invested for the purpose of forming a Nurses' Pension Fund. Many tragic facts relating to the hardships of nurses after their earning period is over have been revealed by the promoters of the Nation's Fund for Nurses in the course of the last two or three years, and their efforts are bearing fruit in many directions. The determination to let the public know the true facts brought a shower of abuse on Lady Cowdray and her coadjutors, but she must be already aware that she has wrought a great change in the public attitude towards the women whose earnings even now are often so pitiably small. We trust that in years to come every town will have its own Nurses' Pension Fund, and that many benefactors will emulate the example of Mr. Watson in assigning some part of their gifts to hospitals to the nurses, without whom such institutions could not exist.

---

The former medical superintendent of the Whitchurch Mental Hospital at Cardiff gives, as we expected, an indignant denial as "grotesquely untrue" to the reports which were circulated at the Cardiff Trades and Labour Council of disorder having taken place under the *régime* of female nurses. Colonel Goodall states that 2,000 cases of mental disorder among the troops were treated with the help of trained certificated sisters, and a staff of nurses and probationers with men to help, and he went on to say: "No one with experience of the presence, example, and influence of trained female nurses and all they stand for with regard to humanity, refinement, and devotion would wish to replace women by men." It is contended, on the other hand, that the worst cases were transferred to Scotch hospitals "to protect the female nurses," but that, nevertheless, "probationary nurses, 17 to 18 years of age, who were not trained nurses, but were learning the business, had been engaged in the work, and they had seen sights which would shock even the depraved women of the streets." The subject is not one which can be usefully discussed on a public platform. It should be settled by persons acquainted with all the facts and qualified to pronounce on them.

---

The managers and matron of the Royal Southern Hospital, Liverpool, are to be warmly congratulated on the re-organisation (recently announced) of their nursing arrangements. In the eyes of nurses, the test of good management lies in liberal salaries. Those at the Royal Southern were considerably augmented at the beginning of the New Year. The Assistant-Matron's salary now rises from £80 to £100 by £5 annually, that of the housekeeping sister from £80 to £90 (in lieu of £70), that of the night sister £65 to £75. Ward sisters, theatre, and out-patient sisters, who formerly received £50 to £55, now have £60 to £70. The Royal Southern knows how to retain its sisters, and nearly all are already entitled to the maximum. The private nursing staff receive £50 and £60 in respective years in lieu of £35 to £40, with £5 bonus. Probationers receive £18, £24, £30, and pass on to the private staff, if suitable for this work, in their fourth year. Indoor uniform is supplied to all, and outdoor uniform also to nurses on the private staff. We are glad to note, also, that a yearly holiday of one month has now been extended to the whole nursing staff. This will affect probationers in their first and second years, who formerly had only two or three weeks' holiday respectively.

---

From time to time complaints of rat-ridden quarters in old institutions make themselves heard. Dr. Howarth's new method of raticide affords the easiest and surest way of exterminating the pests which has yet been brought to notice. Strong lithographic varnish warmed by plunging the container into hot water till the stuff becomes liquid is to be spread thickly on battens of wood or cardboard in the neighbourhood of the runs with an attractive bait in the middle, about an inch being left clear of varnish on the edge. The rats readily climb on after the bait, get stuck and are found dead next morning. The theory is that they die of fright.

---

The admirable work which Miss A. Smith carried through at the Kingston Infirmary has been warmly recognised by the many friends she made during her term of office. The past and present members of her staff have presented her with a valuable set of table cutlery of the new stainless type, a pair of carvers, silver salt spoons, and other

**ROUND THE HOSPITALS—(*continued*).**

table accessories. The domestic staff gave her a cruet-stand, the laundry workers a Wedgewood biscuit box, and the male workers a cake basket. She took a prominent part in re-organising the Infirmary as a training-school for nurses, and brought it to a high pitch of efficiency. During the past ten years the school has turned out 147 nurses, all of whom have done well since. Many distinguished themselves during the war, and some doing fine work as matrons are passing on the lessons of their training-schools. It is indeed impossible to gauge the extent of a good matron's influence on succeeding generations of nurses. Miss Smith has suffered under the system which goads able administrators to overwork, and this has been aggravated by war conditions. We may be permitted to hope that after a prolonged rest her services may be secured in the larger world of nursing under the Registration Board, the College of Nursing, or some of the numerous activities of the Ministry of Health.

---

The nursing staff at the City Hospital, Walker Gate, Newcastle, are very fortunate in the public interest which attaches to their training and the prizes which reward those who come out first in the examinations. The prize-giving was made the occasion of a great revel given to the nurses by Alderman Stableforth, in which over 200 persons were present. The Lord Mayor opened the ball with the Matron, Miss H. E. Cook, and the Lady Mayoress followed with Dr. Kerr, Medical Officer of Health. Nine out of ten nurses passed the examination of the Fever Nurses' Association and received their certificate. The Heath and Carr bequests were divided among the candidates who held the first four places as follows: 1st, Nurse M. E. Lightfoot, £6 and the Stableforth Gold Medal as the best nurse in her year; 2nd, Nurse A. Hutchison, £4 and the Stableforth Silver Medal; 3rd, Nurse G. Wilkinson, £3 (to which a medal was added); 4th, Nurse J. Smith, £1 10s. The training of the nurses in fever hospitals is so often carried out in a hole-and-corner manner, without the smallest public recognition that the proceedings deserve to be chronicled and imitated.

---

According to a report of the Metropolitan Asylums Board, success has attended the system of obtaining recruits for the staff—including nurses at mental hospitals—through the Ministry of Labour. The Board has been able to obtain staff urgently required in greater numbers and at shorter notice than otherwise could be expected. The Board has also, owing to the number of war nurses now being demobilised, been in a position, through the instrumentality of the Nurses' Demobilisation and Resettlement Committee, to divert some of these nurses into the Board's service at a time when nurses were badly needed. Suitable candidates for appointments as assistant nurses and probationers have also been secured.

---

It is perhaps a considerable stretch of imagination definitely to connect, as does a lay contemporary, the recent increase of sensational robberies and acts of personal violence, with after-effects of the war-time air-raids on London. The suggestion raises points, however, which are very noticeable to the practising doctor though not necessarily so obvious to his patients themselves. It is true, as far as crime goes, that a number of the recent convictions have related, almost pathetically, to men of good position and first-class war records. A motive for the act has been practically absent, and medical evidence decidedly in favour of pronouncing the root cause as a nervous system shattered as the result of war strain. The percentage of such instances is at the moment difficult to ascertain, but, coming to ordinary medical care of the people, one notes on every hand how the varied anxieties and strains of the last few years have produced some measure of disturbance in the nervous system of a large proportion of the community. Undoubtedly in making judgment, medical or otherwise, we must remember and allow for these facts. We are now at the reaction period both in health and temperament. Even the wholesale employment of women in ways to which they were both unaccustomed and often most unsuited has been by no means an unmixed blessing to the sex.

---

The Metropolitan Asylums Board has been considering the question as to whether service as a V.A.D. nurse should be allowed to count towards the period of probationership in children's nursing. The point was raised by a recent applicant for appointment as probationer at one of the children's hospitals. She had had two years' continuous service as a member of the V.A.D. in territorial, general and military hospitals, and wished to know whether this was to count for nothing or whether a reduction in the length of probationership could be made. The Board consulted its principal medical officer and the medical superintendents of the children's hospital; and the Institution Committee now reports that it proposes that service of the nature in question should be allowed to count on the following conditions:—(i) Service shall have been rendered in properly equipped military hospitals and not at a convalescent home; (ii) A candidate with not less than two years of such service who undertakes to stay for two years in one of the Board's children's hospitals shall be allowed to sit for the two-years' certificate at the end of her first year under the Board and for the three-years' certificate at the end of her second year; (iii) The ordinary certificate of the Board shall be given to such probationers, a footnote being added to the effect that one year of training has been excused on account of two years' previous work at military hospitals; (iv) The commencing salary shall be that of a second-year probationer.

